# Screening and Characterization of 1,8-Cineole-Based Solvents as an Alternative to Hexane for Obtaining Nonpolar Compounds from Plant-Based Milk Coproducts

**DOI:** 10.1021/acssuschemeng.4c05897

**Published:** 2024-10-17

**Authors:** Monique M. Strieder, Felipe S. Bragagnolo, Jose A. Mendiola, Maurício A. Rostagno, Elena Ibáñez

**Affiliations:** † Foodomics Laboratory, Instituto de Investigación en Ciencias de la Alimentación (CIAL, CSIC-UAM), Madrid 28049, Spain; ‡ Multidisciplinary Laboratory of Food and Health (LabMAS), School of Applied Sciences (FCA), Universidade Estadual de Campinas, Campinas, São Paulo 13484-350, Brazil

**Keywords:** COSMO-RS, eutectic solvents, almond, peanut, fatty acids, agricultural coproducts

## Abstract

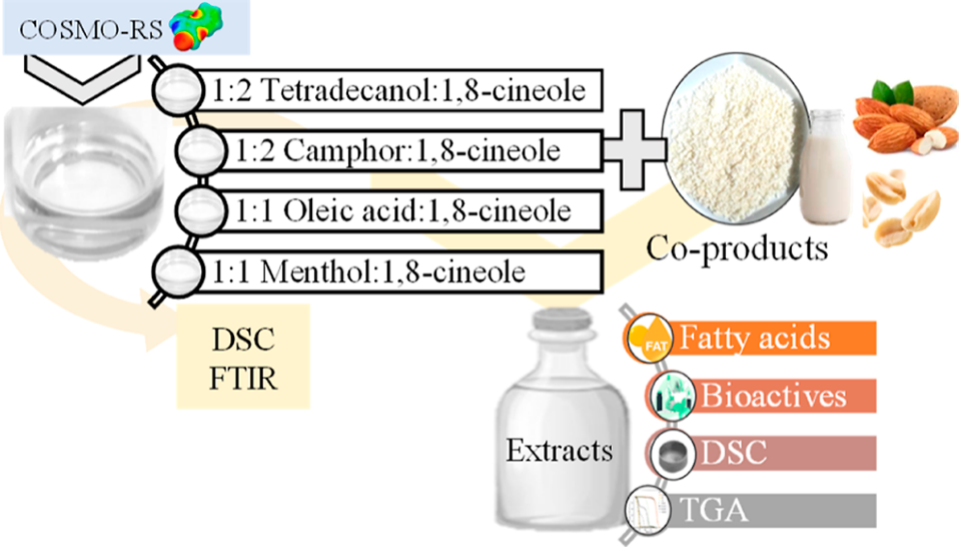

The design of new hydrophobic solvents is essential for replacing the toxic hexane for extracting nonpolar compounds such as fatty acids. On the other hand, the full use of plant matrices seeking to obtain new food and pharmaceutical products from their coproducts has also been the focus of sustainable processes. This study proposed new solvents for replacing hexane to extract fatty acids and hydrophobic bioactive compounds from coproducts obtained from almond- and peanut-based milk processing. The COSMO-RS method was used to select terpene-based mixtures to substitute hexane. Experimentally, four liquid solvents were formed from 1:2 tetradecanol/1,8-cineole (TE/EU), 1:2 camphor/1,8-cineole (CA/EU), 1:1 oleic acid/1,8-cineole (OL/EU), and 1:1 menthol/1,8-cineole (ME/EU). DSC analyses indicated the reduction of the CA/EU, OL/EU, and ME/EU melting points concerning their components. However, the melting point values predicted by the COSMO for obtaining eutectic mixtures differed. CA/EU was the only mixture with a melting point lower than the COSMO-RS-predicted one. In contrast, the FTIR spectra did not provide a clear visualization of the hydrogen bond formation between camphor and 1,8-cineole. This could be due to the formation of weak hydrogen bonds, a phenomenon observed in other studies. Nevertheless, these solvents have the advantage of low viscosity, a promising feature that likely facilitated mass transfer in the extraction of hydrophobic compounds from almond and peanut coproducts. ME/EU provided the same global extraction yield as hexane and higher phytosterol extraction from almond coproducts. On the other hand, CA/EU provided the same global yield and squalene content as hexane from peanut coproducts. The extracts can be directly used in food and pharmaceutical applications since the solvents are usually part of the formulations. However, DSC and TGA-DTA analyses indicated possible ways to separate the solvents.

## Abstract 2

The comprehensive utilization of plant matrices and the exploration of new solvents to replace traditional toxic ones are crucial for advancing sustainable chemistry.

## 1 Introduction

Plant-based milk production is expanding as more consumers are becoming lactose-intolerant, developing milk protein allergies, or choosing plant-based diets.^1,2^ This market is projected to be worth a substantial USD 47.1 billion in 2033, with an estimated annual growth of 8.3% in the next decade (from 2023 to 2033).^3^ The almond-based milk market has already established a strong presence in several countries, while the peanut-based milk market is still growing.^4,5^ However, these two plant matrices share several similarities, including a high fat (∼50 g/100 g) and protein (∼20 g/100 g) content.^6,7^

Considering the significant growth of the plant-based milk industry, Lorente et al. (2023)^8^ pointed out that the solid that remains after obtaining these beverages, a coproduct of the extraction process, has not been studied and should be valorized. This solid material is probably a rich source of oil and bioactive compounds not extracted by water. In this sense, Ouzir et al. (2021)^9^ highlighted the almond oil application in food and pharmaceutical products due to its activity in cardiovascular risk management, oxidative stress reduction, neuroprotection, and as an antioxidant agent. These activities were associated with its vitamin E (including tocopherols and tocotrienols), phytosterols, and squalene content. Vitamin E acts as a free radical scavenger, protecting the oil against lipid oxidation; phytosterols are known for reducing plasma low-density lipoprotein cholesterol, while squalene has been presenting vital oxygen scavenging and antitumor activities. Peanut oil, likewise, is characterized by a rich composition of tocopherols and unsaturated fatty acids that have beneficial effects on metabolism.^10^

Hexane is the solvent most widely used to obtain soybean, cottonseed flaxseed, safflower seed, and other seed oils due to its efficiency in extracting hydrophobic compounds.^11^ However, this solvent is flammable, toxic, and nonbiodegradable and may be found in traces in final products.^12^ Thus, the growing search to replace petroleum-based solvents with green ones has proposed hydrophobic deep eutectic solvents (DES) prepared with naturally occurring terpenoids as alternatives.^13,14^ These solvents present low toxicity, high biodegradability, stability, and tunability, allowing ready-to-use applications.^15^ In this regard, previous research by Rodrigues et al. (2020)^16^ evaluated terpene-based eutectic solvents to recover astaxanthin from brown crab shell residues, demonstrating combined bioactivity between extracted compounds and solvents to inhibit colorectal cancer cells and bacterial growth.

Computational tools, such as the COnductor-like Screening MOdel for Real Solvent (COSMO-RS), have been valuable approaches for selecting alternative solvents for extracting hydrophobic compounds.^14,17^ Cao and Su (2021)^18^ have also provided a list of natural compounds that could be used to form these solvents. However, few studies have reported the characterization of these solvents, aiming to observe the formation of eutectic mixtures, understand their properties, and propose separation possibilities. Thus, analyzing solvents’ functional groups, phase transitions, and thermogravimetric characteristics can add detailed information about these solvents and extracted compounds.

This study proposes an almond and peanut valorization, evaluating and characterizing 1,8-cineole (eucalyptol)-based solvents predicted by COSMO-RS as replacements for hexane in the extraction of fatty acids and bioactive compounds. This approach aims to provide new, sustainable ingredients for the food and pharmaceutical industries while contributing to the ongoing scientific exploration of green solvents.

## 2 Materials and Methods

### 2.1 Chemicals

1,8-Cineole (eucalyptol) (TCI, >98% purity, CAS: 470–82–6), menthol (Alfa Aesar, 99% purity, CAS: 89–78–1), camphor (Sigma, 95% purity, CAS: 200–945–0), oleic acid (VWR Chemicals, 79% oleic acid, 12% linoleic acid, 4% palmitic acid, 2% stearic acid, and ∼1% palmitoleic acid, CAS: 112–80–1), 1-tetradecanol (TCI, >98% purity, CAS: 112–72–1), and borneol (Sigma, 87% borneol and 12% isoborneol) were the components employed to formulate solvents. The organic solvents cyclopentyl methyl ether—CPME—(VWR Chemicals, 99.9% purity, CAS: 5614–37–9) and hexane (VWR Chemicals, mixture of isomers, CAS: 110–54–3) were also employed to perform the extraction. Almonds and peanuts in shells were purchased from Casa Ruiz Granel Selecto (Madrid/ES). According to the supplier, peanuts contain 49.2, 25.8, 16.1, and 8.5 g/100 g of fat, proteins, carbohydrates, and fiber, while almonds have 55, 20.5, 6, and 11.5 g/100 g of fat, proteins, carbohydrates, and fiber, respectively.

### 2.2 Solvent Selection by COSMO-RS and Preparation

The COSMO-RS method was employed to select terpene-based solvents to replace hexane. Geometry optimization and molecular charge density analysis for the COSMO-RS assessments were performed using Turbomole software (TmoleX 2022 version 22.0.0) under the COSMO-BP-TZVP specification. These calculations were conducted using the COSMOtherm package (version 22.0.0) with the BP_TZVP_22 settings. For the purpose of solvent screening, 595 samples were generated by pairing 35 natural constituents suggested by Cao and Su (2021)^18^ (Table S1). Combinations of each compound were chosen based on the infinite dilution activity coefficient values (ln γ^∞^) having the potential to be the most effective for replacing hexane. The four compositions (1-tetradecanol/1.8-cineole, camphor/1.8-cineole, 1.8-cineole/oleic acid, and 1.8-cineole/borneol) presenting the lower activity coefficient values were experimentally tested, in the beginning, in the molar proportion of 1:1 (w/w). Terpene-based solvents were prepared by weighing component mass to acquire a molar ratio of 1:1 in a capped reagent bottle and heated from 60 to 100 °C under magnetic stirring until no insoluble compounds were observed. Solvents were stored for 24 h to verify their stability.

### 2.3 Terpene-Based Solvent Characterization

#### 2.3.1 Density and Viscosity

Solvent densities were measured in duplicate by using a 10.548 cm^2^ pycnometer. Solvent density, viscosity, and melting temperature were also predicted using a platform to search the physicochemical properties of eutectic solvents developed by Odegova et al. (2024).^19^ Measurements and predictions were performed at 25 °C.

#### 2.3.2 Fourier Transform Infrared Spectroscopy

The Fourier transform infrared (FTIR) spectrum of terpene-based solvents and their individual components were obtained using an FTIR spectrometer (FTIR Bruker ifs66v) at room temperature and recorded in the 450–4000 cm^–1^ range.

#### 2.3.3 Differential Scanning Calorimetry

DSC analysis of all terpene-based solvents efficiently synthesized as well as their starting constituents were evaluated in a Q100 DSC system (TA Instruments) in the temperature range from −40 to 200–300 °C, depending on the sample at 10 °C/min, after equilibration for 3 min at −40 °C. The analyses were performed under a nitrogen atmosphere (50 mL/min), with 2.57–9.47 mg of samples in aluminum pans covered with lids.^20^

Solid–liquid equilibrium (SLE) diagrams for the terpene-based solvents were predicted by COSMO-RS, employing melting points determined by DSC for the components (1,8-cineole, 1-tetradecanol, camphor, oleic acid, and menthol).^21^ The enthalpy and entropy of fusion values used for calculations were as follows: 10.5 and 0.0368 kJ/mol·K for 1,8-cineole, 47.1 and 0.146 kJ/mol·K for 1-tetradecanol, 6.8 and 0.02025 kJ/mol·K for camphor, 33.4 and 0.286 kJ/mol·K for oleic acid, and 19.87 and 0.0535 kJ/mol·K for menthol.

### 2.4 Plant-Based Milk Coproduct and Its Characterization

Almond and peanut-based milk were prepared in the laboratory to obtain coproducts from this process. About 500 g of almonds and peanuts in the shell were soaked in water at 50% (500 g of oilseeds for 1 L of water) for 20 h at room temperature (24 ± 2 °C). After the soaking time, the oilseeds’ hydration was 43 and 59% (w/w) for almonds and peanuts, respectively. The seeds were peeled, and the plant-based milk preparations were obtained in batches using a relation ratio of 2:1 (w/w) of ultrapure water/hydrated and peeled almonds or peanuts in a Thermomix (500 W, Vorwerk) for 3 min^22^. After the plant-based milk extraction, the solid material, the raw material of this study, was separated, lyophilized, and stored at −24 °C.

Freeze-dried almond and peanut solid materials were characterized according to their Sauter mean diameter and moisture, fat, and protein content. The Sauter mean diameter was determined using sieves from 0.25 to 2 mm. The moisture was measured in a DAB moisture analyzer (Kern), using 1 g of the raw material. The protein content was determined by the Kjeldahl method, using a conversion factor of 6.25. The fat content was determined by Soxhlet extraction, employing hexane for 6 h.

### 2.5 Solid–Liquid Extractions

Solid–liquid extractions were performed in a Fisherbrand Multi-Platform Shaker at 5000 rpm and room temperature (25.2 °C) for 10 min using a S/F of 10 (w/w), weighing 1 g of almond or peanut coproduct and 10 g of each solvent. The experiments were performed in duplicate for each of the eight solvents, four terpene-based solvents, their two liquid components (1.8-cineole and oleic acid), and as control hexane and CPME. The global yield of the extractions was determined by drying 0.5 g of each extract at 80 °C under N_2_ at a pressure of 20 psi.

### 2.6 Gas Chromatography

The fatty acid profile of extracts was determined by gas chromatography in a Shimadzu GC 2010 gas chromatography system (Kyoto, Japan) equipped with a Shimadzu AOC-20i autosampler injector coupled to a QP-2010 Plussingle quadrupole mass spectrometer. The derivatization of the compounds in their methyl esters was performed according to Golmakani et al. (2014)^23^ by employing 30 mg of each liquid extract in duplicate. The column was a ZB-WAX, 30 m × 0.25 mm i.d. fused silica capillary column with a 0.25 μm film thickness (Phenomenex, USA). The injector, interface, and ionization chamber temperature levels were maintained at 240, 245, and 250 °C, respectively. A gradient oven temperature programing starting at a 2 min hold at 30 °C with a ramp of 20 °C/min to 180 °C with a 2 min hold at 180 °C, a ramp of 4 °C/min to 207 °C with a 3 min hold at 207 °C, and another ramp of 2 °C/min to 220 °C was applied for the separation of fatty acid methyl esters (FAMEs), according to the Xu et al.’s (2010)^24^ methodology. A 1 μL sample was injected into the GC–MS system with the injector in split-less mode. Helium was used as the carrier gas. A solvent delay of 2.5 min was selected for MS. A Shimadzu GC Solution software was used to process the data. Compounds were detected by mass spectrometry in the SCAN mode using a mass interval ranging from 40 to 400 *m*/*z* and then identified by comparing their mass spectra with an FAMEs/d27-Myristic acid mixture standard (Agilent Fiehn GC/MS Metabolomics, USA) and with the Wiley Library.

Other volatile compounds were also analyzed in the same gas chromatograph using a ZB-5plus, 30 m × 0.25 mm i.d. fused silica capillary column with a 0.25 μm film thickness (Phenomenex, USA). The injector, interface, and ionization chamber temperature levels were maintained at 250, 335, and 250 °C, respectively. A gradient oven temperature programing starting at 45 °C with a ramp of 10 °C/min to 290 °C and a ramp of 5 °C/min to 325 °C with a 15 min hold at 325 °C was applied for the separation of compounds. Undiluted liquid extracts were injected into the GC–MS system with the injector in split mode (1:10). The identification and quantification of α-tocopherol in almond coproduct extracts was confirmed using a standard calibration curve from 0.0625 to 1 mg/mL (*R*^2^ = 0.9618). The same curve was employed to quantify other bioactive compounds as equivalents of α-tocopherol.

### 2.7 Thermal Analysis

Thermogravimetric analyses (TGA) of extracts acquired in hexane, CPME, terpene-based solvents, EU, and OL were performed using a Q500 V6.7 Build 203 TGA instrument (TA Instruments). The analyses were performed by heating the sample (8.45 mg to 57.2 mg) from 30 to 700 °C, at a rate of 10 °C min^–1^, under a flow of nitrogen (20 mL/min). DSC analyses were also performed according to Section 2.3.3 for almond and peanut oil acquired by hexane extraction, according to Section 2.5 after evaporating the solvent.

### 2.8 Solvents’ Impact on the Sustainability of Extraction Processes Using Path2Green

The Path2Green metric was employed to verify proposed solvents as alternatives to hexane in terms of their effects on the environmental, social, and economic aspects. This analysis generates a score from −1 to 1 considering the study of biomass, transport, pretreatment, solvent, scaling, purification, yield, post-treatment, energy, application, repurposing, and waste management of extraction processes.^25^

### 2.9 Statistical Analysis

The mean difference was verified by analysis of variance using the Minitab 18 software with a 95% confidence level (*p*-value ≤0.05). Tukey’s test of means was performed at a 95% confidence level (*p*-value ≤0.05).

## 3 Results and Discussion

### 3.1 Terpene-Based Solvent Selection and Formation

The solvent selection was assessed by evaluating their activity coefficients at infinite dilution (ln γ^∞^) across hexane employing COSMO-RS. Combinations that presented lower activity coefficients concerning hexane were 1-tetradecanol/1,8-cineole (0.19), camphor/1,8-cineole (0.24), oleic acid/1,8-cineole (0.28), and borneol/1.8-cineole (0.29). Thus, formulations using these four combinations were experimentally prepared at a molar ratio of 1:1. Table 1 presents the heating conditions under which a homogeneous liquid phase was achieved, together with their visual appearance right after formulation and after 24 h of storage at room temperature (25 ± 5 °C).

**Table 1:**
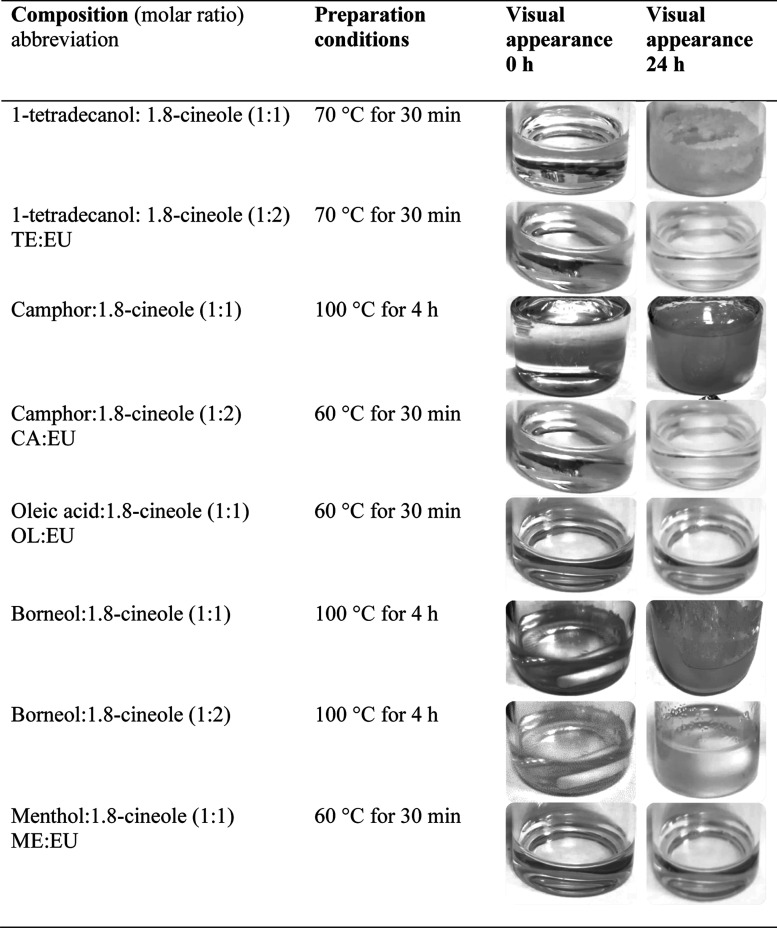
Terpene-Based Solvent Preparation Conditions and Stability after 24 h

All mixtures formed a homogeneous liquid after stirring and heating (from 60 to 100 °C) (Table 1). However, after 24 h of storage at room temperature, the mixtures of 1-tetradecanol/1.8-cineole, camphor/1.8-cineole, and borneol/1.8-cineole presented recrystallized solid materials or were solidified at room temperature. In this case, the compound’s molar composition did not allow for reaching a liquid solvent and the eutectic point. The eutectic point represents the chemical composition where the melting point of a mixture of two components, interacting mainly through hydrogen bonds, is lower than its components.^26^ Therefore, the molar ratio of 1:1 did not allow us to obtain a liquid solvent with 1-tetradecanol/1.8-cineole, camphor/1.8-cineole, and borneol/1.8-cineole, and other molar fractions were tested.

In this sense, a higher proportion of 1.8-cineole was added, using a molar ratio of 1:2, with the aim of obtaining a liquid solvent at room temperature (∼25 °C). This higher 1.8-cineole proportion allowed the formation of a stable liquid solvent with 1-tetradecanol and camphor. However, the 1:2 molar ratio of borneol/1.8-cineole also did not allow for obtaining a liquid solvent. The following mixture that presented lower activity coefficients concerning hexane, menthol/1.8-cineole (0.32), was chosen to substitute the borneol/1.8-cineole solvent. This solvent was prepared in a molar ratio of 1:1. Therefore, the mixtures that remained liquid after 24 h of storage were 1:2 tetradecanol/1.8-cineole (TE/EU), 1:2 camphor/1.8-cineole (CA/EU), 1:1 oleic acid/1.8-cineole (OL/EU), and 1:1 menthol/1.8-cineole (ME/EU).

**Figure 1:**
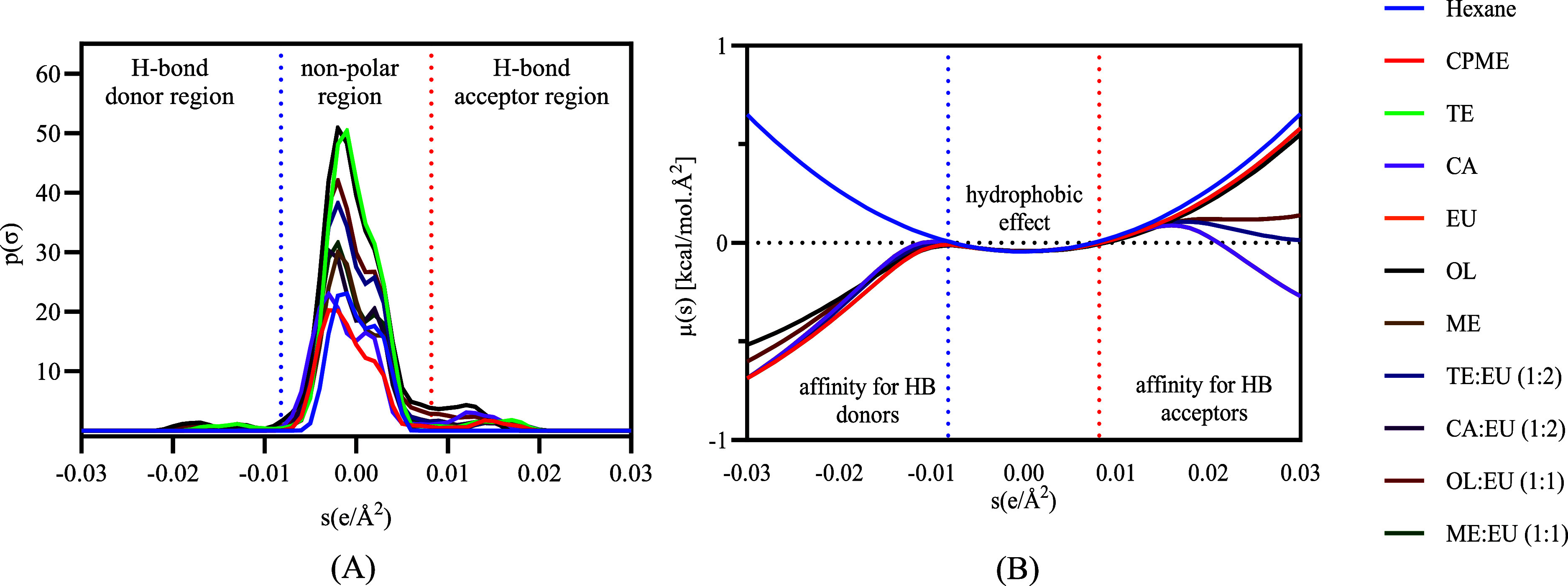
COSMO-RS-generated (A) σ-profiles and (B) σ-potentials for hexane, CPME, formulated terpene-based solvents, and their individual components.

Figure 1 presents the σ-profiles (Figure 1A) and σ-potentials (Figure 1B) acquired for these mixtures, their individual components, hexane, and CPME by COSMO-RS.^27^ The σ-profiles are divided into three regions: hydrogen bond donors (HBD), nonpolar, and hydrogen bondacceptor (HBA). Thus, the σ-profile of solvents and pure compounds is similar, presenting nonpolar characteristics, given their peaks are around 0 e/A^2^. In contrast, the σ-potential indicates the affinity of components and solvents for the HBA, nonpolar interactions, and HBD, respectively. A different behavior was observed since hexane presented a higher affinity for nonpolar components, as indicated by the parabola centered at 0 e/A^2^ compared to the other solvents. All other components and solvents showed a higher affinity for HBD compounds than hexane. CA, TE/EU, and OL/EU presented a higher affinity for HBA than others. Therefore, the affinity of CA for HBA and of EU for HBD can indicate the formation of a eutectic mixture or a deep eutectic mixture through hydrogen bonds between these two components. DES are formed when the eutectic point of a mixture of two or more pure compounds occurs at a temperature lower than that of an ideal liquid mixture, exhibiting significant negative deviations from ideal behavior.^28^ The formation of this type of mixture normally occurs through the use of HBA and HBD components, which form strong hydrogen bond interactions.

On the other hand, the similar σ-potential observed for the different components (EU, TE, OL, and ME) may indicate the nonformation of a eutectic or a deep eutectic mixture but rather a mixture that is liquid at room temperature (∼25 °C). Fan et al. (2021)^29^ proposed that terpene–terpene mixtures form weak-to-medium hydrogen bonds through electrostatic interactions, which in turn are associated with low viscosities. Additionally, CPME, OL, and the formulated solvents are likely to extract a broader range of compounds with varying polarities due to their higher affinity for HBD than hexane.

### 3.2 Terpene-Based Solvent Characterization

Solvent densities were measured and predicted using the platform developed by Odegova et al. (2024)^19^ for comparison purposes. Viscosities and melting temperatures were also predicted by employing the same platform. Table 2 presents these results and additional information about the control solvents.

**Table 2:** Measured Density and Predicted Density, Viscosity, and Melting Point of Solvents

	measured (25 °C)	predicted (25 °C)		
solvent	density (g/mL)	density (g/mL)	viscosity (cP)	melting temperature (°C)
TE/EU	0.879 ± 0.001	0.97	3.6	–15
CA/EU	0.930 ± 0.001	0.91	4.3	–9
OL/EU	0.899 ± 0.001	0.99	5.2	7
ME/EU	0.907 ± 0.001	0.91	4.5	–33
Hexane	0.66^a^		0.3^a^	–95^a^
CPME	0.86^a^		0.549^b^	–140^a^
EU	0.92^a^		2.58^c^	2^a^
OL	0.89^a^		15.3^d^	11–14^a^

a Informed by the supplier.

b Randová et al. (2018).^30^

c Nikolic et al. (2020).^31^

d Yamawaki (2023)^32^ at 40 °C.

All terpene-based solvents presented a higher density than hexane and CPME. CA/EU presented the highest density, followed by ME/EU, OL/EU, and TE/EU. This result was expected because when the molecules interact by hydrogen bonds, they compress the closest molecules, increasing their density.^33^ On the other hand, hexane has a lower density because molecules are larger and do not form strong hydrogen bonds, occupying a higher volume. The prediction efficiently determined the CA/EU and ME/EU densities. In contrast, predicted values showed a difference of approximately 0.1 g/mL concerning the measured densities of TE/EU and OL/EU. In the case of the prediction for OL/EU, the difference between the measured and predicted densities may be related to the purity of the OL, which contains other fatty acids that can interact differently with EU.

The predicted values for the viscosities of 1,8-cineole-based solvents were much higher than those of hexane and CPME, which may affect mass transfers during extraction and the pumping of solvents in industrial processes. However, high viscosity may benefit specific applications, such as developing ready-to-use extracts for the cosmetics industry.^15^ Moreover, predicted viscosities showed much lower values than those observed for hydrophilic DESs (from 9 to 11 cP).^34^

### 3.3 Differential Scanning Calorimetry

**Figure 2:**
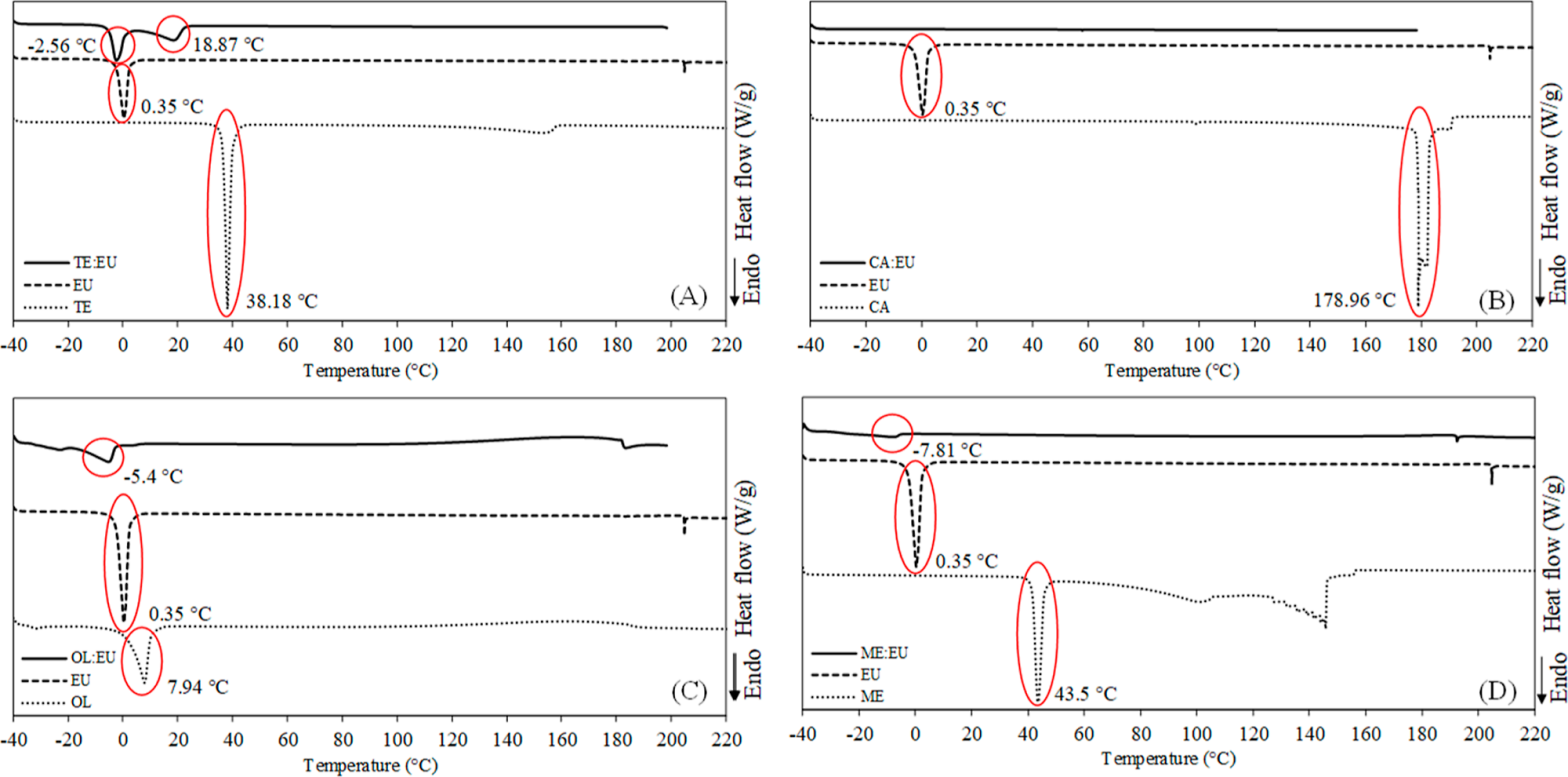
DSC thermograms obtained for solvents and their individual components: (A) TE (1-tetradecanol), EU (1,8-cineole), and TE/EU (1:2); (B) CA (camphor), EU, and CA/EU (1:2); (C) OL (oleic acid), EU, and OL/EU (1:1); (D) ME (menthol), EU, and ME/EU (1:1).

Figure 2 presents 1,8-cineole-based solvents and their components’ thermograms demonstrating their phase transitions. Most formulated solvents presented lower melting points than their pure components (highlighted in red in Figure 2). The lower melting points acquired for CA/EU, OL/EU, and ME/EU concerning their pure components indicated the formation of a eutectic mixture at the studied molar proportions (Figure 2B–D). The melting point of CA/EU was reduced to below −40 °C, so its peak was not observed under the analysis conditions. On the other hand, the melting point of the TE/EU (Figure 2A) was reduced considering the TE, but two melting peaks were observed for this mixture, suggesting the presence of two melting regions. Moreover, one of the melting points was observed at a higher temperature than the one observed for EU. Eutectic mixtures presented just one melting point at a lower temperature than their components.

The formation of a eutectic or deep eutectic solvent through DSC analysis of only a molar proportion of components of a mixture cannot be confirmed. In this sense, the COSMO-RS was used to predict SLE diagrams between 1,8-cineole-based solvent components. These results are presented as the Supporting Information (Figure S1).

The formation of a eutectic mixture between TE and EU was predicted at a molar composition of 0.077 TE to 0.922 EU, and this mixture would have a melting point of −4.2 °C (Figure S1A). Experimentally, a liquid mixture was observed at room temperature (∼25 °C) employing a molar composition of 0.333 TE to 0.666 EU, presenting two melting points at −2.56 and 18.87 °C (Figure 2A). Therefore, TE/EU cannot be considered a eutectic mixture but rather a liquid solvent at temperatures higher than 18.87 °C. In contrast, CA/EU demonstrated characteristics of a deep eutectic solvent. COSMO-RS predicted the formation of a eutectic mixture in the molar ratio of 0.158 CA and 0.842 EU, presenting a melting point of −9.15 °C (Figure S1B), while the experimentally tested mixture employing 0.333 CA to 0.666 EU, presented a melting point at a lower temperature than −40 °C (Figure 2B). Thus, a significant negative deviation from ideal behavior was observed, probably indicating the formation of a deep eutectic solvent.

A eutectic mixture at the molar proportion of 0.275 OL and 0.725 EU, presenting a melting temperature of −48.44 °C (Figure S1C), was predicted. In comparison, a solvent with molar proportions of 0.500 OL and 0.500 EU was formulated, presenting a melting point of −5.4 °C (Figure 2C). In this case, as observed for TE/EU, the molar proportions employed probably did not achieve a eutectic mixture. COSMO predicted the formation of a eutectic mixture between ME and EU at a molar composition of 0.238 ME to 0.762 EU, and this mixture would have a melting point of −15.54 °C (Figure S1D). In contrast, the prepared solvent at a molar relation of 0.500–0.500 allowed a melting point of −7.81 °C, probably also not reaching the eutectic condition.

Moreover, the significant difference observed between the predicted melting points (Table 2) and those obtained by DSC analysis (Figure 2) at the same molar proportions clearly indicates the need for further research. In this study, despite not confirming the formation of eutectic mixtures, the formulations were used as solvents since they remained liquid after 24 h and under the extraction temperature.

### 3.4 FTIR Spectroscopy

**Figure 3:**
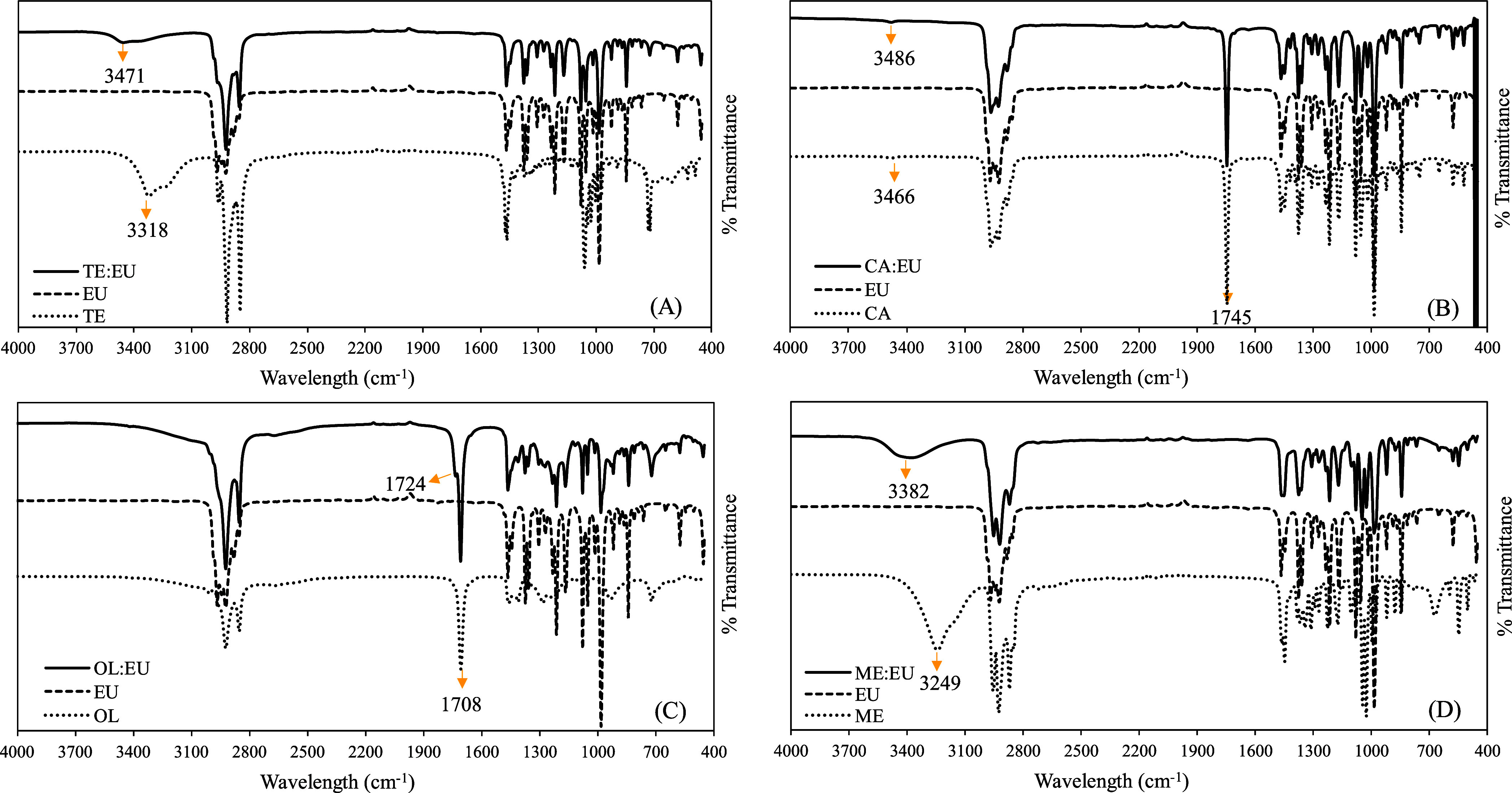
FTIR spectra obtained for solvents and their individual components: (A) TE (1-tetradecanol), EU (1,8-cineole), and TE/EU (1:2); (B) CA (camphor), EU, and CA/EU (1:2); (C) OL (oleic acid), EU, and OL/EU (1:1); (D) ME (menthol), EU, and ME/EU (1:1).

FTIR spectra of the terpene-based solvents and their components are presented in Figure 3. The lower-wavenumber region (1600–400 cm^–1^) presents the spectral features of the pure compound’s functional groups. In contrast, the 3800–3000 cm^–1^ region is dominated by the OH contributions, where it is possible to observe the formation of hydrogen bonds between the components.^35^ In this sense, O–H stretching vibrations were observed in the spectra obtained for TE, CA, ME, TE/EU, CA/EU, and ME/EU (Figure 3A,B,D). Peaks associated with OH stretching were observed for TE, CA, and ME at centered wavelengths of 3318, 3466, and 3249 cm^–1^, respectively. The stretching of O–H observed for TE and ME was more intense and at a lower wavelength than the ones observed for TE/EU and ME/EU. This modification may indicate the formation of hydrogen bonds between the compounds to form the terpene-based solvent of TE/EU and ME/EU. Rodrigues et al. (2020)^16^ observed similar spectral differences by analyzing the pure compounds and the solvent formed with ME/EU and ME/CA (1:1), indicating the formation of hydrogen bonds between the compounds.

CA and CA/EU presented similar and less intense peaks in the region of 3400 cm^–1^. The low intensities of the OH vibrations observed for CA and CA/EU make it difficult to confirm the formation of hydrogen bonds between the components. Fan et al. (2021)^29^ have proposed that terpene–terpene DES form weak-to-medium hydrogen bonds through electrostatic interactions, which in turn are associated with low viscosities. This concept could have significant implications, suggesting that CA/EU, with its low predicted viscosity (Table 2) and minimal changes in FTIR spectra due to hydrogen bond formation, could also exhibit similar properties.

Furthermore, in the region of approximately 1700 cm^–1^ the vibration of the C=O group can also demonstrate shifts in its stretching frequency due to the formation of hydrogen bonds with O–H groups.^36^ A peak was observed for OL at 1708 cm^–1^, representing the stretching of the C=O bond of its carboxyl group.^37^ The intensity of this vibration was modified in the spectrum obtained for OL/EU and may also indicate the formation of hydrogen bonds between the compounds.

### 3.5 Almond and Peanut Hydrophobic Compound Extraction and Characterization

**Figure 4:**
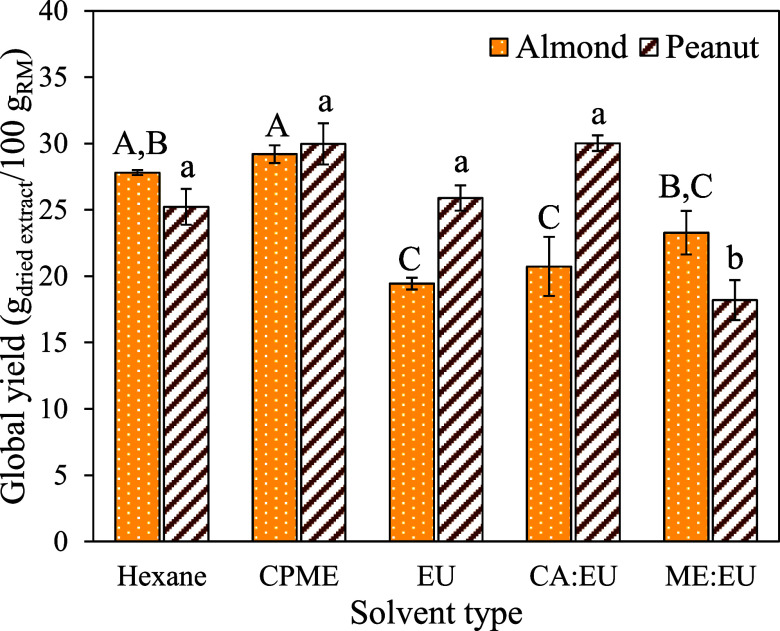
Effect of solvents on the global extraction yield of the hydrophobic compounds from almond and peanut coproducts. Values followed by different uppercase or lowercase letters show differences by Tukey’s test at 95%.

The freeze-dried almond and peanut coproducts presented 0.71 ± 0.01 and 0.88 ± 0.03 mm Sauter mean diameters, 3.0 ± 0.1 and 4.9 ± 0.5 g/100 g moisture contents, 56 ± 1 and 44 ± 1 g/100 g fat contents, and 23.4 ± 0.4 and 24.4 ± 0.1 g/100 g protein contents, respectively. Thus, this coproduct’s potential as a source of hydrophobic compounds was verified. Extractions were performed with the four developed solvents (TE/EU, CA/EU, OL/EU, and ME/EU) and with hexane, CPME, EU, and OL as control solvents. After extracting the hydrophobic compounds from almond and peanut coproducts, the global yield was determined by volatilizing the solvents from an aliquot of the extracts. Figure 4 presents the results acquired for the global yield after the volatilization of solvents.

It is worth mentioning that the volatilization of TE/EU, OL/EU, and OL under the same conditions used for the other solvents did not allow for total solvent remotion. The terpene-based solvent that allowed higher extraction yield from almond coproduct was ME/EU, followed by CA/EU. On the other hand, CA/EU presented the same efficiency as hexane, CPME, and EU for extracting hydrophobic compounds from peanut coproducts. The differences observed concerning solvent efficiency for extracting bioactive compounds from almond or peanut coproducts may be associated with the availability of compounds in these plant matrices, affecting mass transfer. Peanut coproduct compounds are probably more available, facilitating convective extraction by either solvent. On the other hand, almond compounds could be less available, requiring more energy for mass transfer. In this case, a less viscous solvent, such as hexane and CPME has an advantage because the homogenization promoted during extraction facilitates greater solvent–compound interaction.

The overall yield after attempting to evaporate OL, EU/OL, and TE/EU was the same for the solvents and extracts (solvents plus extracted hydrophobic compounds). The solvent and extract (solvent + extracted compounds) after trying to evaporate the solvent showed 34 ± 0.3, 66 ± 1, and 100 g/100 g of global yield for TE/EU, OL/EU, and OL, respectively. Therefore, evaporation conditions (80 °C under nitrogen flow) possibly allowed the volatilization of EU, which has a lower boiling temperature (176 °C) than that of OL (286 °C) and TE (266 °C). In this sense, using these solvents makes quantifying and determining their efficiency challenging. On the other hand, ME/EU and CA/EU allowed global yields more similar to hexane, showing their potential as an alternative to this toxic solvent.

### 3.6 Fatty Acid Profile and Bioactive Compound Content

Terpene-based solvents did not affect the fatty acid profile of the extracted almond and peanut hydrophobic compounds. However, the profile of fatty acids extracted by OL/EU and OL was not determined because it was impossible to separate the solvent’s oleic acid from the extract’ oleic acid, causing saturation of the chromatogram that hampered adequate identification/quantification of compounds. The fatty acid profile determined for each extract is presented in Table 3.

**Table 3:** Effect of Solvents on the Fatty Acid Profile of Almond and Peanut Compounds^a^

	fatty acids (% area)					
solvent	palmitic	palmitoleic	stearic	oleic	linoleic	eicosenoic
Almond Extracts						
hexane	14 ± 1^a^	1.3 ± 0.1^a^	1.8 ± 0.2^b^	63 ± 1^a^	20.1 ± 0.3^a^	nd
CPME	14 ± 1^a^	1.2 ± 0.1^a^	1.7 ± 0.1^a^	63 ± 1^a^	20 ± 1^a^	nd
EU	14 ± 1^a^	1.2 ± 0.1^a^	1.7 ± 0.1^a^	64 ± 1^a^	19 ± 1^a^	nd
OL	na	na	na	na	na	na
TE/EU	15 ± 1^a^	2 ± 1^a^	1.6 ± 0.2^a^	61 ± 1^a^	19.6 ± 0.1^a^	nd
CA/EU	16 ± 1^a^	1.4 ± 0.1^a^	1.4 ± 0.1^a^	62 ± 1^a^	18.7 ± 0.3^a^	nd
OL/EU	na	na	na	na	na	na
ME/EU	16 ± 1^a^	1.5 ± 0.1^a^	1.5 ± 0.1	61 ± 1^a^	19.1 ± 0.1^a^	nd
Peanut Extracts						
hexane	11 ± 1^a^	nd	1.7 ± 0.1^a^	80 ± 2^a^	5 ± 1^a^	2.0 ± 0.2^a^
CPME	12 ± 1^a^	nd	1.5 ± 0.1^a^	81 ± 1^a^	3.7 ± 0.3^a^	1.8 ± 0.2^a^
EU	12 ± 1^a^	nd	1.7 ± 0.1^a^	81 ± 2^a^	4 ± 2^a^	1.6 ± 0.1^a^
OL	na	na	na	na	na	na
TE/EU	10 ± 2^a^	nd	1.5 ± 0.3^a^	81 ± 2^a^	4.8 ± 0.4^a^	1.5 ± 0.1^a^
CA/EU	13 ± 2^a^	nd	1.5 ± 0.2^a^	81 ± 1^a^	3.5 ± 0.2^a^	1.6 ± 0.4^a^
OL/EU	na	na	na	na	na	na
ME/EU	12 ± 2^a^	nd	1.6 ± 0.3^a^	81 ± 1^a^	4 ± 1^a^	1.6 ± 0.3^a^

a Values followed by different letters in the same column show differences by Tukey’s test at 95%. nd: nondetected and na: nonanalyzed.

The main fatty acids determined in almond extracts were oleic and linoleic acids, followed by palmitic, stearic, and palmitoleic acids. A similar profile was determined for almond (*Prunus amygdalus* L.) varieties from Turkey by Beyhan et al. (2011)^38^ and for *Prunus dulcis* by Askin et al. (2007).^39^ The oleic/linoleic acid ratio above 3 is within the expected range for almond oil obtained directly from the seed.^40^ This ratio value demonstrates the oil’s oxidative stability since oleic acid is more stable than linoleic acid. Furthermore, obtaining plant-based milk did not alter the expected profile of almond oil fatty acids, showing that this coproduct can be a good source of these fatty compounds. Different from what was observed for almonds, peanut hydrophobic fractions presented oleic acid as the primary fatty acid, followed by palmitic, linoleic, eicosenoic, and stearic acids. In this sense, this peanut variety was produced with high-oleic genes, presenting about an 80% oleic acid content, an oleic/linoleic acid ratio of approximately 20, and consequently extended shelf life.^41^ Like almonds, peanut coproducts have a high potential for obtaining fatty acids.

**Figure 5:**
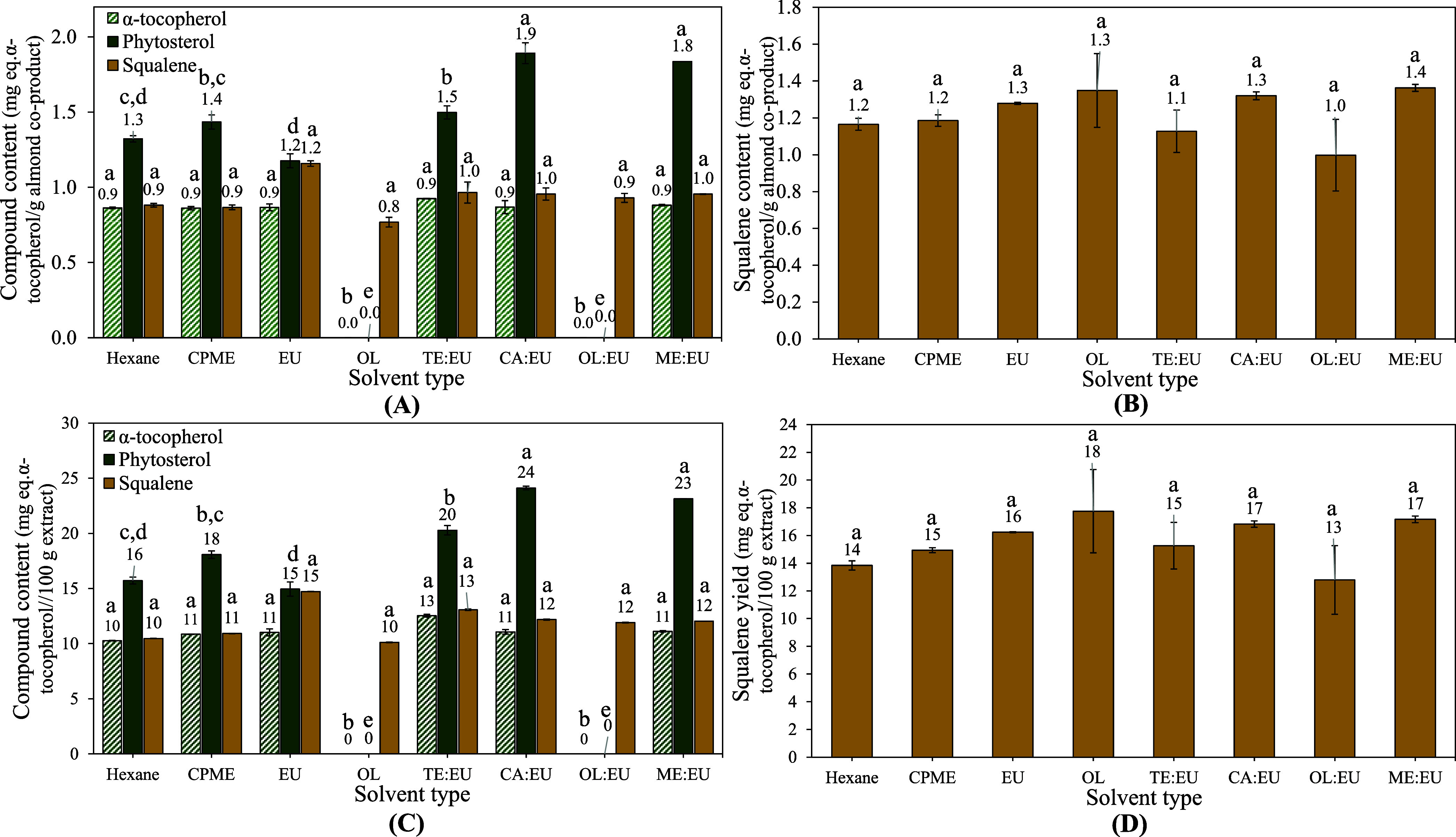
Solvent effect on the extraction of bioactive compounds from (A,C) almond and (B,D) peanut coproducts.

Figure 5 presents the effect of solvents on the extraction of α-tocopherol, squalene, and a phytosterol (tentatively identified as campesterol) expressed in milligram equivalents of α-tocopherol/g of coproducts (Figure 5A,B). Extraction yields were also expressed in milligram equivalents of α-tocopherol/100 g of extract (Figure 5C,D), considering the possibility of applying the extracts obtained in terpene-based solvents directly to food and pharmaceutical products. A-tocopherol and the phytosterol were not identified in almond extracts acquired with OL/EU and OL (Figure 5A,C). CA/EU and ME/EU allowed for higher extraction of phytosterol than the other solvents. On the other hand, squalene content was not affected by the solvent type.

The differences observed toward the extraction of phytosterol may be due to solvents’ σ-potentials and physical parameters; for instance, CA/EU and ME/EU presented similar affinity for HB donors and an equal density, higher than that of hexane, CPME, and OL and lower than for OL/EU, TE/EU, and EU (Table 2). Moreover, these solvents presented similar predicted viscosity. Squalene was the only bioactive compound identified in extracts obtained from peanut coproducts. Moreover, the type of solvent had no significant effect (*p*-value ≥0.05) on the compound extraction. In this sense, terpenoid-based solvents showed similar or higher efficiency for extracting hydrophobic bioactive compounds than organic solvents (hexane and CPME).

### 3.7 Thermal Characteristics of Almond and Peanut Hydrophobic Extracts

**Figure 6:**
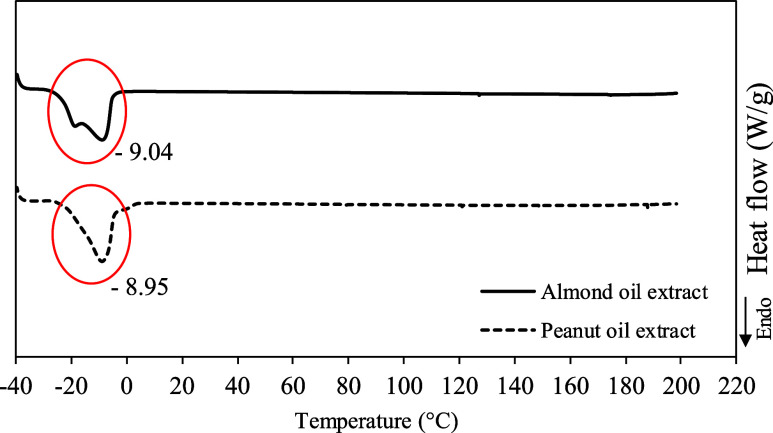
DSC thermograms of almond and peanut extracts.

DSC thermograms of almond and peanut oil extracts were also analyzed to observe the melting points of the extracted hydrophobic compounds. Figure 6 shows similar melting points for almond and peanut hydrophobic compounds around −9.04 and −8.95 °C, respectively.

The proposal to use terpene-based solvents would be to obtain ready-to-use extracts. However, when it is necessary to concentrate the extract, these thermal results, which suggest a more appropriate way to separate the compounds from the solvents, may be of particular interest. Comparing DSC thermograms for TE/EU, almond, and peanut compounds (Figure S2A) indicated that TE/EU could be partially separated by cooling the sample to about −5 °C (Figure S2A). At this temperature, TE/EU finishes to solidify, while almond and peanut compounds remain in a liquid state. Thus, the liquid fraction, almond or peanut extract, could be separated. On the other hand, separating compounds extracted with CA/EU could be carried out by cooling the sample below −30 °C since almond and peanut compounds finish solidifying at temperatures of −24.13 and −19.82 °C, respectively (Figure S2B). Thus, the remaining solid fraction (almond and peanut compounds) could be separated from the solvent that would remain a liquid. Separating the hydrophobic almond and peanut compounds from OL/EU and ME/EU by cooling would be difficult since they have similar melting regions.

**Figure 7:**
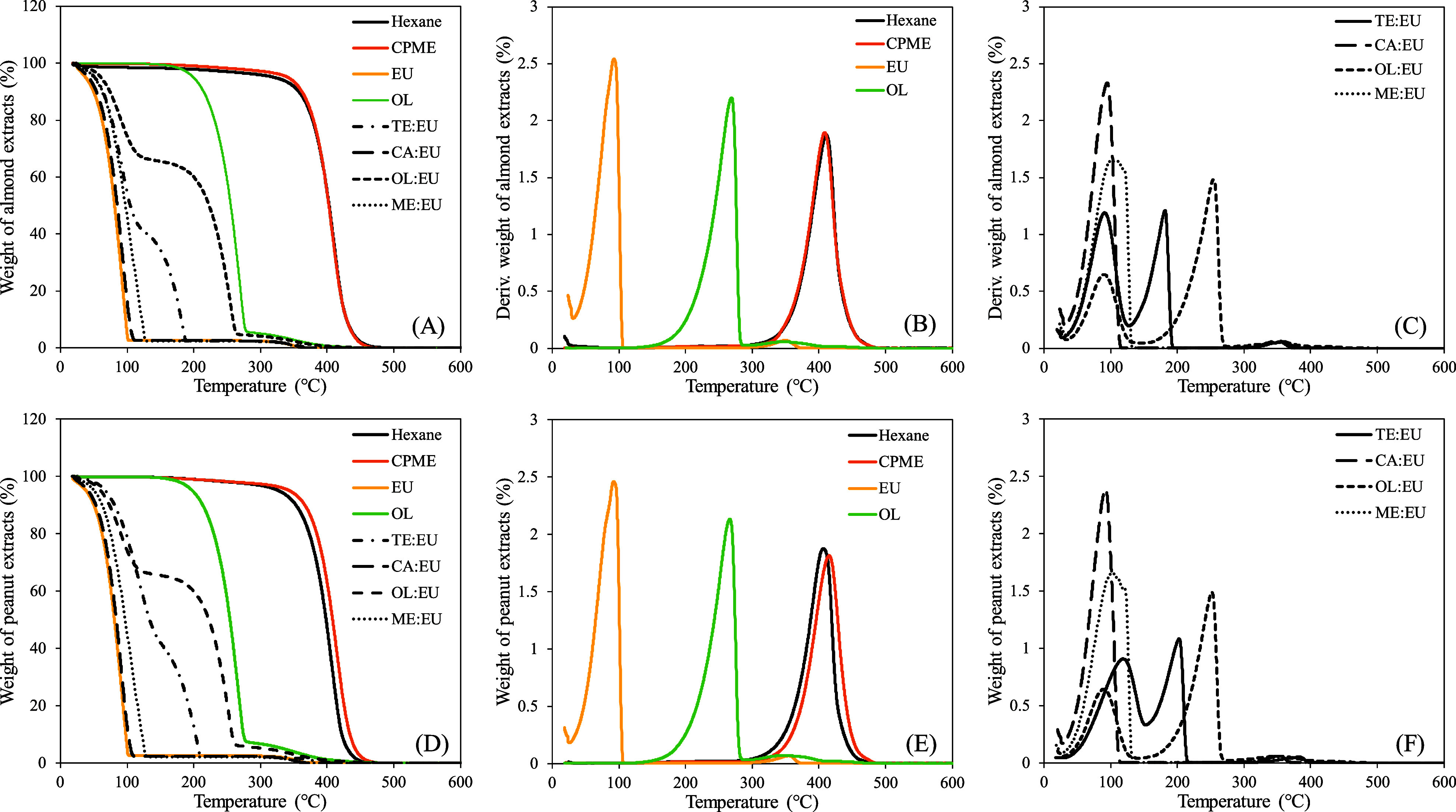
TGA curves acquired for (A) almond and (D) peanut compounds in different solvents and (B,C,E,F) their TGA–DTA.

Thermogravimetry and differential thermal analysis (TGA–DTA) of the extracts allowed the verification of solvent separations involving volatilization. Figure 7 presents the TGA–DTA curves for almond and peanut hydrophobic compounds in the different solvents. First, it is noteworthy that the extracts obtained in hexane and CPME present TGA and TGA–DTA only from hydrophobic compounds extracted from almonds and peanuts (Figure 7A,B,D,E). Moreover, the region with the highest thermal degradation of almond and peanut compounds between 300 and 500 °C was observed in all extracts. Therefore, the volatilization of EU, CA/EU, and ME/EU at 100 °C does not interfere with the mass reduction of hydrophobic compounds extracted from almonds and peanuts.

On the other hand, only a fraction of TE/EU and OL/EU was thermally degraded at around 100 °C that can be due to the volatilization of EU; this observation confirms our previous results (Section 3.4); Figure 7C,F indicates that the final mass loss of tetradecanol appears to be around 200 °C. The OL final degradation appears at 275.7 °C, but this peak was followed by lower ones between 300 and 400 °C. In this case, after 300 °C, it would be difficult to determine the amount of oleic acid from the solvent or hydrophobic compounds extracted from almond and peanut coproducts.

### 3.8 Environmental, Social, and Economic Impacts of Extraction Solvents

**Figure 8:**
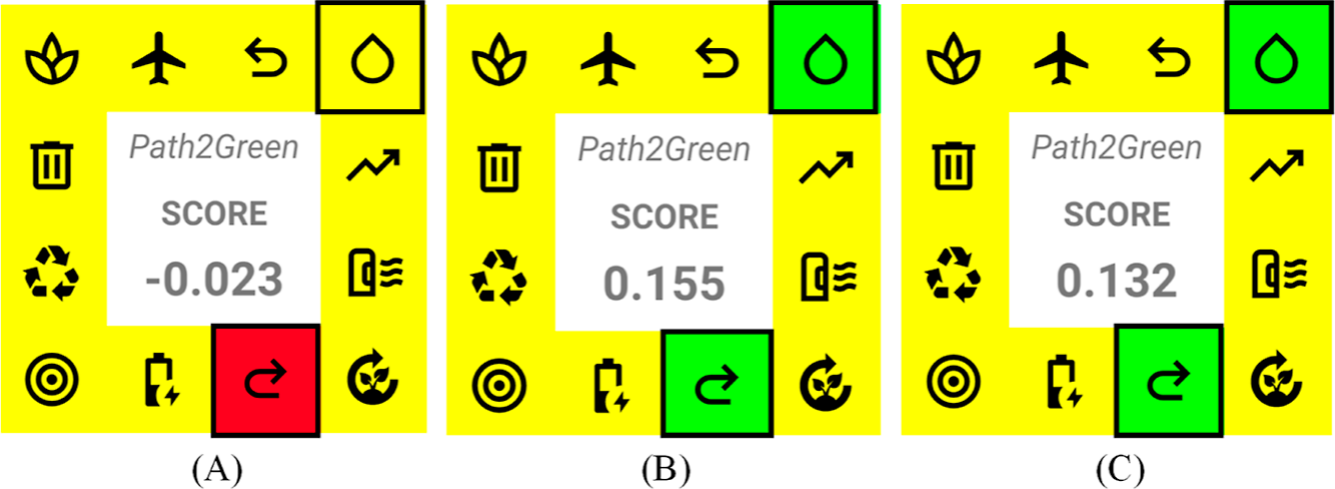
Scores of processes employing (A) hexane/CPME, (B) 1,8-cineole-based solvents considering extracts ready-to-use, and (C) up to two steps of post-treatment.

The choice of the extraction solvent mainly affects two of the 12 principles considered for evaluating an extraction process regarding environmental, social, and economic impacts by the Path2Green metric: the solvent and the post-treatment of the extract.^25^Figure 8 presents the scores obtained for the extraction processes considering only these two parameters by the Path2Green metric.

The choice of solvent significantly impacts environmental and social issues. A nontoxic and biodegradable solvent makes the extraction process safe for the environment and workers. Among solvents recommended for extraction, hexane and CPME were considered problematic due to their high volatility and contaminant emissions, which present high environmental impact and health risks. In this sense, hexane and CPM solvent processes were scored with 0 for the solvent principle (Figure 8A). On the other hand, nontoxic compounds from renewable sources are recommended for the formulation of new solvents, scored with +1. The terpene-based solvents evaluated in this study not only fall within the recommended solvents but also hold great potential for future solvent selection.

1,8-Cineole or eucalyptol is acquired from eucalyptus oil and is safe at normal doses, showing an IC50 of 4 mM for the HCT 116 cell line.^42,43^ 1-Tetradecanol is a long-chain fatty alcohol derived from tetradecanoic acid, presents low toxicity, and is naturally sourced from coconut or palm oil.^44^ Camphor is naturally produced from the bark of *Cinnamomum camphora* L. trees, presenting a toxicity LD_50_ of 9487 mg/kg in male rats and an IC50 of 4.5 mM for the HCT 116 cell line.^43,45^ Oleic acid is found in animal and vegetable oils and has been associated with positive effects on human health, especially by helping to treat cardiovascular diseases.^46^ Menthol is a monoterpene extracted from *Mentha canadensis* L. and M. × piperita L., presenting cooling characteristics as one of the most important flavorings.^47^ The 50% inhibitory concentration of menthol for the cellular and subcellular systems ranged from 0.32 to 0.76 mM.^48^ Therefore, for the solvent principle of the Path2Green metric, the 1,8-cineole-based solvents were scored with +1 (Figure 8B,C).

In the post-treatment stage, the solvent also significantly affects the environmental and economic aspects, especially when it is problematic (volatile, nonbiodegradable, and toxic) and needs to be eliminated. In the hexane and CPME processes, the solvent must be evaporated using physical procedures. Thus, according to the metric, the post-treatment of these processes is scored at −0.50, considering combining two post-treatments based on problematic solvents. On the other hand, the best scenario is to obtain ready-to-use extracts without eliminating the solvent, scored as +1 (Figure 8B). The solvents and extracted compounds achieved in this study could be used together due to the following solvent characteristics:^42,45,47^

- 1,8-Cineole presents antimicrobial and anti-inflammatory action.
- 1-Tetradecanol is used as a chemical intermediate, emulsion stabilizer, fragrance, and viscosity-increasing agent in plasticizers, antifoam agents, soaps, perfumes, detergents, suppositories, creams, shampoos, toothpaste, and cleaning preparations.
- Camphor is used as an anticold and antiseptic agent, a source of fragrance in cosmetic products, a scent in household products, a flavoring in foods, and an intermediate in the production of chemicals used for making perfumes.
- Oleic acid has been used as an ingredient in food formulations as a substitute for saturated fats, maintaining normal blood cholesterol, modulating inflammatory markers, blood pressure, and others.
- Menthol is used in confections such as chocolate and chewing gum, oral-care products, and medicinal products for its cooling and biological effects.

However, depending on the application, it may be necessary to remove the solvent or concentrate the compounds; in this case, the processes using 1,8-cineole-based solvents would be scored at +0.5 (Figure 8C). A negative score for the extractive process using hexane and CPME was observed through the metric that evaluated the solvent effect on environmental, economic, and social aspects. On the other hand, positive scores were observed using the terpene-based solvents proposed in this study, demonstrating their potential for improving the sustainability of extractive processes.

## 4 Conclusions

Four different 1,8-cineole-based solvents were prepared and characterized as an alternative to hexane for extracting hydrophobic compounds from almond and peanut coproducts. DSC analysis indicated the reduction of OL/EU, CA/EU, and ME/EU melting points concerning its components. CA/EU was the only mixture with a melting point lower than the COSMO-RS-predicted one. However, a clear response regarding the formation of hydrogen bonds between CA and EU was not detected through FTIR analyses. Concerning extraction performance, ME/EU allowed the same overall yield as hexane in extracting hydrophobic compounds from almonds, while CA/EU allowed the same yield as hexane in extracting hydrophobic compounds from peanuts. The solvent type did not affect the fatty acid profile of the almond and peanut extracts. Moreover, the coproducts showed good sources of these compounds, presenting a fatty acid profile similar to that expected for the seeds. ME/EU allowed a higher phytosterol content, demonstrating an efficiency higher than that of hexane for extracting this bioactive compound. The extracts can be directly used in food and pharmaceutical applications since the solvents are natural and are usually part of formulations. However, DSC and TGA provided insights into separating the extract from the solvent. Finally, the analysis of the solvents’ effect on the environmental, social, and economic impact of extraction processes demonstrated the potential wins for sustainability using 1,8-cineole-based solvents instead of hexane and CPME.

## Supporting Information Available

Natural constituents selected for screening solvents, SLE diagrams predicted by COSMO-RS, and DSC thermogram comparison (PDF)
