# Supplementary material for: Screening and Characterization of 1,8-Cineole-Based Solvents as an Alternative to Hexane for Obtaining Nonpolar Compounds from Plant-Based Milk Coproducts
Source: ACS Sustain Chem Eng. 2024 Oct 17;12(43):16052–63. doi: 10.1021/acssuschemeng.4c05897 (PMC11524417; doi:10.1021/acssuschemeng.4c05897)
Supplement: Supplementary file 1 — sc4c05897_si_001.pdf [file sc4c05897_si_001.pdf]

Supporting Information (SI)

Screening and characterization of 1,8-cineole-based solvents as an alternative to hexane for obtaining nonpolar compounds from plant-based milk co-products

Monique M. Strieder [a,b]*, Felipe S. Bragagnolo [b], Jose Mendiola [a], Maurício A. Rostagno [b]*, Elena Ibáñez [a]*

[a] Foodomics Laboratory, Instituto de Investigación en Ciencias de la Alimentación (CIAL, CSIC-UAM), Madrid, 28049, Spain

[b] Multidisciplinary Laboratory of Food and Health (LabMAS), School of Applied Sciences (FCA), Universidade Estadual de Campinas, São Paulo-SP, 13484-350, Brazil

*Corresponding authors: mms@unicamp.br, mauricio.rostagno@fca.unicamp.br, and elena.ibanez@csic.es

Number of pages: 5

Number of figures: 2

Number of tables: 1

I. Natural constituents selected for screening solvents……………………………………(S2)

II. SLE diagrams by COSMO-RS for solvents components…………………………………(S4)

III. DSC thermograms comparison……………………………………………………….(S5)

I. Natural constituents selected for screening solvents

Table S1. Natural constituents selected for screening solvents by COSMO-RS.

Chemical name	Molar mass (g/mol)	Molecular formula
1,2-Decanediol	146.227	$C_{10}H_{22}O_2$
1-Naphthol	144.17	$C_{10}H_8O$
1-Tetradecanol	214.39	$C_{14}H_{30}O$
Acetic acid	60.052	CH_3COOH
Anisyl alcohol	138.16	$C_8H_{10}O_2$
Arginine	174.2	$C_6H_{14}N_4O_2$
Atropine	289.369	$C_{17}H_{23}NO_3$
Borneol	154.25	$C_{10}H_{18}O$
Butyric Acid	88.11	$C_4H_8O_2$
Camphor	152.23	$C_{10}H_{16}O$
Coumarin	146.1427	$C_9H_6O_2$
Decanoic acid	172.26	$C_{10}H_{20}O_2$
Decanol	158.28	$C_{10}H_{22}O$
Dodecanoic acid	200.3178	$C_{12}H_{24}O_2$
Eucalyptol (1,8-cineole)	154.249	$C_{10}H_{18}O$
Hexanoic acid	116.1583	$C_6H_{12}O_2$
Lactic acid	90.08	$C_3H_6O_3$
Lauric acid	200.3178	$C_{12}H_{24}O_2$
Leucine	131.17	$C_6H_{13}NO_2$
Levulinic acid	116.11	$C_5H_8O_3$
Lidocaine	234.3373	$C_{14}H_{22}N_2O$
Menthol	156.27	$C_{10}H_{20}O$
Myristic acid	228.37	$C_{14}H_{28}O_2$
Nonanoic acid	158.23	$C_9H_{18}O_2$
Octanoic acid	144.21	$C_8H_{16}O_2$
Octanol	130.2279	$C_8H_{18}O$
Oleic acid	282.46	$C_{18}H_{34}O_2$
Palmitic acid	256.43	$C_{16}H_{32}O_2$
Perillyl alcohol	152.237	$C_{10}H_{16}O$

Pyruvic acid	88.06	$C_3H_4O_3$
Serine	105.09	$C_3H_7NO_3$
Thymol	150.22	$C_{10}H_{14}O$
Benzyl alcohol	108.14	C_7H_8O
Pyrazole	68.07	$C_3H_4N_2$
Imidazole	68.077	$C_3H_4N_2$

II SLE diagrams by COSMO-RS for solvents components

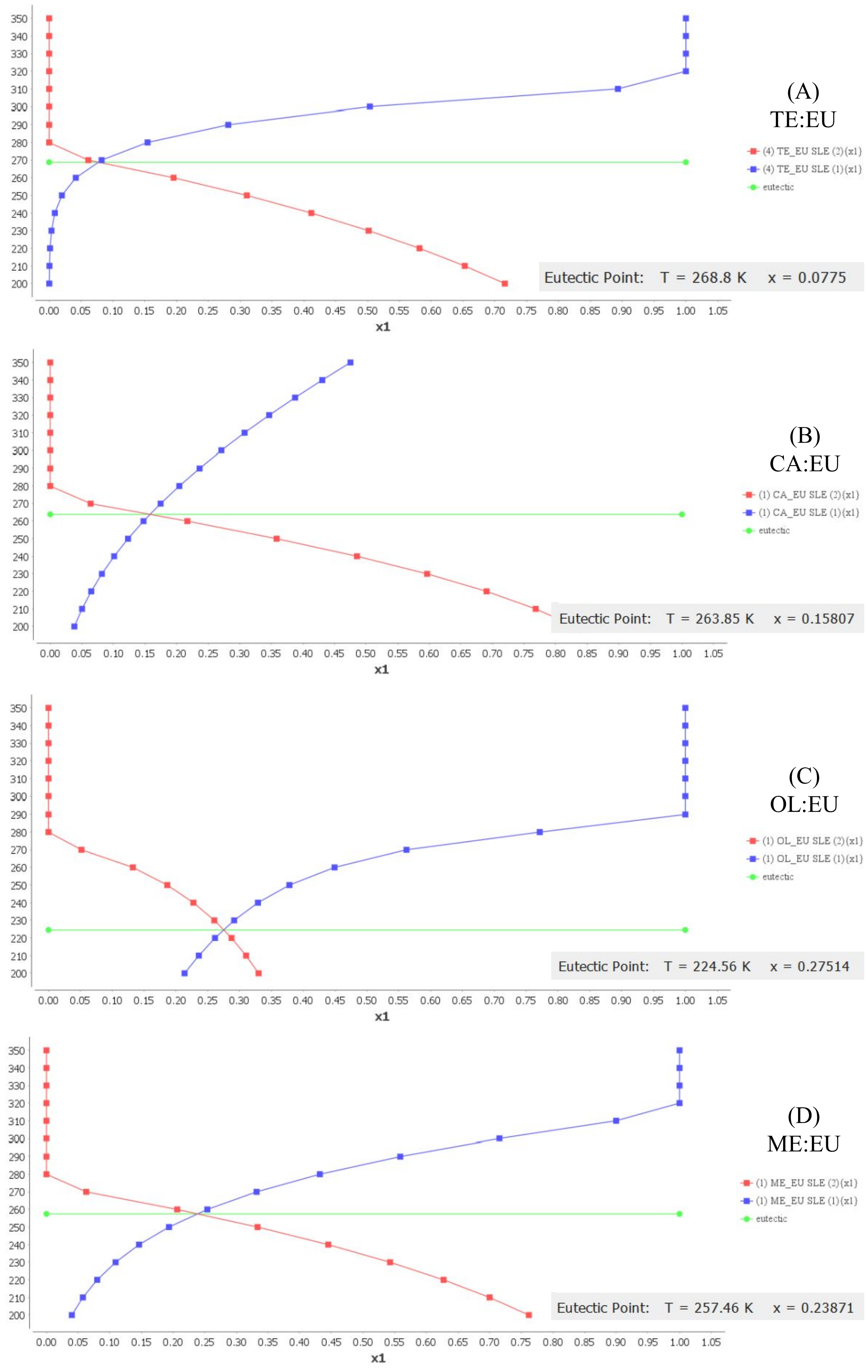

Figure S1. SLE diagrams for (A) TE:EU, (B) CA:EU, (C) OL:EU, and (D) ME:EU.

III. DSC thermograms comparison

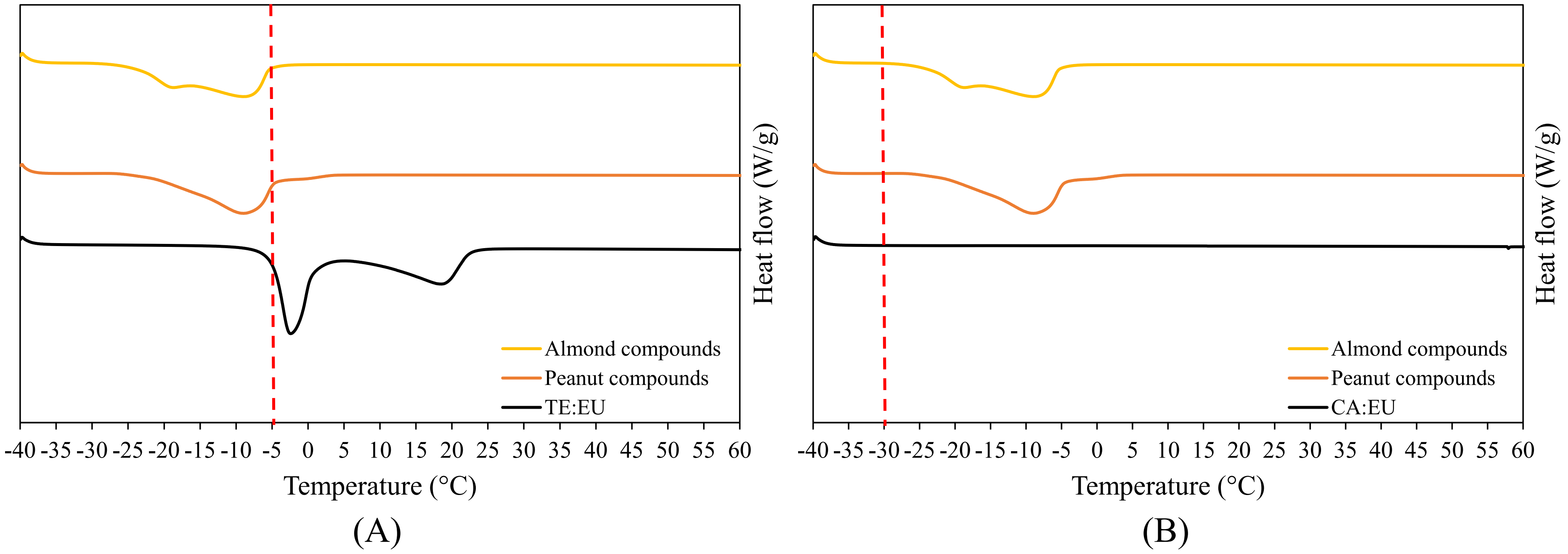

Figure S2. DSC thermograms of almond and peanut extracts compared to (A) TE:EU and (B) CA:EU solvents.
